# Enhanced Garlic Crop Identification Using Deep Learning Edge Detection and Multi-Source Feature Optimization with Random Forest

**DOI:** 10.3390/s25196014

**Published:** 2025-09-30

**Authors:** Junli Zhou, Quan Diao, Xue Liu, Hang Su, Zhen Yang, Zhanlin Ma

**Affiliations:** 1 Henan Institute of Remote Sensing, Zhengzhou 450000, China; 18337190983@163.com (X.L.); 18796213259@163.com (H.S.); yz906711872@163.com (Z.Y.); 2 School of Surveying and Land Information Engineering, Henan Polytechnic University, Jiaozuo 454000, China; diaoquan@gmail.com; 3 School of Surveying and Urban Spatial Information, Henan University of Urban Construction, Pingdingshan 467000, China; hpumzl@163.com

**Keywords:** garlic identification, deep learning, edge detection, feature optimization, field constraint, multi-source remote sensing, precision agriculture

## Abstract

Garlic, as an important economic crop, plays a crucial role in the global agricultural production system. Accurate identification of garlic cultivation areas is of great significance for agricultural resource allocation and industrial development. Traditional crop identification methods face challenges of insufficient accuracy and spatial fragmentation in complex agricultural landscapes, limiting their effectiveness in precision agriculture applications. This study, focusing on Kaifeng City, Henan Province, developed an integrated technical framework for garlic identification that combines deep learning edge detection, multi-source feature optimization, and spatial constraint optimization. First, edge detection training samples were constructed using high-resolution Jilin-1 satellite data, and the DexiNed deep learning network was employed to achieve precise extraction of agricultural field boundaries. Second, Sentinel-1 SAR backscatter features, Sentinel-2 multispectral bands, and vegetation indices were integrated to construct a multi-dimensional feature space containing 28 candidate variables, with optimal feature subsets selected through random forest importance analysis combined with recursive feature elimination techniques. Finally, field boundaries were introduced as spatial constraints to optimize pixel-level classification results through majority voting, generating field-scale crop identification products. The results demonstrate that feature optimization improved overall accuracy from 0.91 to 0.93 and the Kappa coefficient from 0.8654 to 0.8857 by selecting 13 optimal features from 28 candidates. The DexiNed network achieved an F1-score of 94.16% for field boundary extraction. Spatial optimization using field constraints effectively eliminated salt-and-pepper noise, with successful validation in Kaifeng’s garlic.

## 1. Introduction

Garlic represents a globally significant cash crop, possessing substantial culinary value alongside recognized medicinal properties, and holds a prominent position within the global agricultural production system [1]. The accurate acquisition of garlic cultivation area information is crucial for agricultural resource allocation, market forecasting, and food security assurance [2]. As the world’s largest garlic producer, China accounts for 49.66% of the global total cultivation area, with an annual production exceeding 20 million metric tons, playing a pivotal role in the global garlic supply chain [3]. However, traditional methods for surveying crop planting areas, primarily reliant on statistical reports and field surveys, are constrained by limitations including high costs, low efficiency, and poor timeliness, making them increasingly inadequate for meeting the practical demands of modern precision agriculture [4].

Significant progress has been made in recent years regarding the application of remote sensing technology for crop identification and monitoring. Early research primarily relied on single-sensor data and simple spectral features for classification [5], which, while validating the feasibility of remote sensing in agricultural applications, often yielded suboptimal accuracy due to the complexity and similarity of crop spectral signatures [6,7].

To address these limitations, researchers have increasingly explored multi-source data fusion techniques to enhance classification robustness. Belgiu and Csillik [8] achieved promising results in crop mapping by employing a time-weighted dynamic time warping (TWDTW) method with Sentinel-2 data, demonstrating the effectiveness of temporal pattern analysis in distinguishing crop types. This temporal dimension has proven particularly valuable, as evidenced by Li et al. [9], who proposed a crop classification method for the Hexi region utilizing Sentinel-2 multi-temporal data and U-Net deep learning architecture to attain high-precision crop identification. Furthermore, the integration of multiple sensor types has shown remarkable potential. The synergistic potential of combining different sensor modalities has been further demonstrated by Chen et al. [10], who introduced an automated garlic mapping framework integrating Sentinel-1 SAR and Sentinel-2 optical imagery, achieving 95.34% overall accuracy through multi-source time-series data fusion on the Google Earth Engine platform. Tian et al. [3] reinforced these findings with comprehensive research on garlic and winter wheat identification in North China using both active and passive satellite imagery.

The success of these multi-source approaches has coincided with rapid advances in deep learning technologies, which have revolutionized remote sensing image processing capabilities. Zhong et al. [11] established the effectiveness of neural networks for handling time-series remote sensing data through pioneering a deep learning-based multi-temporal crop classification method. The robust capabilities of Convolutional Neural Networks (CNNs) in image recognition and semantic segmentation tasks have proven particularly valuable [12,13], with Lu et al. [14] developing a multi-scale feature fusion semantic segmentation model (MSSNet) that achieved excellent performance in crop classification using high-resolution imagery. Recent architectural innovations have pushed these capabilities further, exemplified by Wang et al. [15], who introduced Cropformer—a Transformer-based approach that demonstrated enhanced generalization capabilities across multiple crop classification scenarios.

Alongside deep learning innovations, traditional machine learning methods continue to play a vital role in crop classification research. Particularly, ensemble methods like Random Forest have significantly enhanced the automation level and identification accuracy of classifications [16,17]. The systematic optimization of feature selection has gained increasing attention, with Yang et al. [5] proposing an optimal feature selection-based crop classification method using a hybrid CNN-RF network, comparing traditional techniques like Random Forest Feature Importance (RF-FI) and Random Forest Recursive Feature Elimination (RF-RFE). Object-based methodologies have also shown promise, as demonstrated by Ma et al. [18], who implemented garlic identification using both active and passive remote sensing data combined with object-oriented approaches.

Despite these advances, the critical importance of incorporating spatial structural information into agricultural applications has become increasingly apparent. Xu et al. [19] addressed this need through their multi-task learning approach for cultivated land field extraction, combining semantic segmentation with edge detection to better define agricultural production units. The methodological foundation for such spatial constraints has been strengthened by Zhang et al. [20], who conducted comprehensive comparative studies of edge detection methods including BDCN, DexiNed, and PidiNet for farmland boundary extraction, identifying DexiNed as particularly effective in agricultural contexts. Li et al. [21] demonstrated in their crop field boundary extraction research that DexiNed exhibits excellent boundary awareness capabilities and can detect more potential field boundaries.

Nevertheless, existing methodologies face several limitations that constrain their practical application. Traditional pixel-based classification methods generate “salt-and-pepper” noise, producing spatially fragmented outputs that inadequately represent actual agricultural production units [9]. Feature selection strategies often lack systematic approaches, with redundant features increasing computational complexity and potentially degrading accuracy [5]. Current deep learning approaches, while powerful in feature extraction, frequently overlook agricultural fields as basic production units. Additionally, boundary extraction accuracy in complex landscapes remains suboptimal [19], and effective spatial constraint mechanisms for integrating field-level information require further development.

Building upon an in-depth analysis of current research and technological trends, this study proposes an integrated framework for precise garlic cultivation area identification in Kaifeng City—a major garlic-producing region in Henan Province, China. The primary contributions of this research are as follows:

1. Deep Learning-Based Boundary Extraction: Implementation of a DexiNed network on high-resolution imagery to accurately extract and vectorize agricultural field boundaries, addressing spatial fragmentation issues in traditional pixel-based crop classification approaches.
2. Optimized Multi-Source Feature Fusion: Integration of Sentinel-1 SAR and Sentinel-2 optical time-series data to construct comprehensive feature sets encompassing polarimetric, spectral, vegetation index, and textural characteristics, with systematic feature selection combining Random Forest Importance and Recursive Feature Elimination techniques.
3. Field-Level Spatial Constraints: Incorporation of extracted agricultural boundaries as spatial constraints to refine pixel-level classification results and generate field-aligned garlic distribution maps that align with actual agricultural production units.
4. Comprehensive Framework Validation: Demonstration of the integrated approach’s effectiveness in achieving high mapping accuracy, reliable multi-source data integration, and practical applicability for precision agriculture applications in complex agricultural landscapes.

## 2. Materials and Methods

### 2.1. Study Area

**Figure 1:**
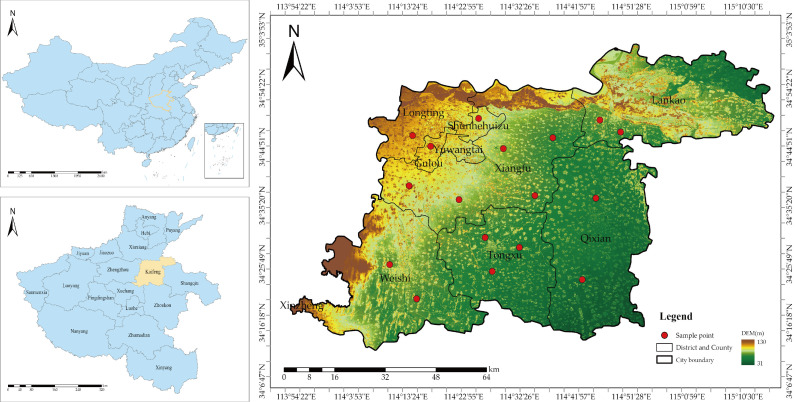
Overview of the research area.

As shown in Figure 1, Kaifeng City (34°12′–35°01′ N, 113°52′–115°15′ E) is located in the central-eastern part of Henan Province, situated in the hinterland of the Huang-Huai-Hai Plain, with a total area of 6444 km^2^. The region is predominantly characterized by Yellow River alluvial plains, accounting for 92.3% of the total area, with an average elevation of approximately 65 m and relatively flat, open terrain. The soil type is mainly fluvo-aquic soil with organic matter content ranging from 1.2% to 1.8%, pH values between 7.5 and 8.0, and water-holding capacity of 35 ± 5 mm/m, demonstrating significant nutrient and water retention capabilities. The climate is classified as warm temperate continental monsoon, with annual mean temperatures of 14.0–14.5 °C. During the garlic growing season (October to May of the following year), the accumulated temperature reaches approximately 2850 ± 50 °C·d, precipitation totals 280 ± 40 mm, and the frost-free period extends for 210–220 days. This climatic configuration creates a unique agricultural thermal-moisture synergistic pattern that provides an optimized environment for winter–spring crop cultivation.

**Figure 2:**
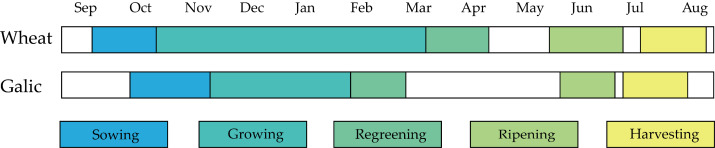
Depiction of the vegetation calendar of the major crops in the study area.

In terms of agricultural structure, garlic and winter wheat constitute the primary cropping systems, with garlic cultivation being particularly prominent. According to 2024 statistics, the city’s garlic cultivation area reached 46.56 kha (accounting for 35% of Henan Province’s total garlic production), establishing it as a national-level agricultural product geographical indication protection zone and one of China’s largest intensive garlic production regions in northern China. Garlic is sown in October, undergoes the seedling stage from November to January of the following year, enters the re-greening stage in February with rapid growth thereafter, matures in June, and is harvested in July. Winter wheat cultivated simultaneously in the study area exhibits similar phenological periods to garlic; therefore, garlic identification requires careful differentiation from winter wheat. The growth cycles of both crops are illustrated in Figure 2.

### 2.2. Sentinel-1/2 Data

Sentinel-1 is a C-band synthetic aperture radar (SAR) satellite constellation consisting of the 1A/1B dual satellites. Its onboard SAR sensor operates at 5.405 GHz, providing radar data in both VV and VH polarization modes with a spatial resolution of 10 m. Sentinel-2 is a high-resolution multispectral imaging satellite constellation consisting of the A/2B dual satellites, with a revisit period of 5 days. Its onboard multispectral imager (MSI) covers 13 spectral bands from the visible to the shortwave infrared, with spatial resolutions of 10 m (four bands), 20 m (six bands), and 60 m (three bands). Detailed band information for the Sentinel-2 sensor is shown in Table 1. The data used in the study are Level-2A surface reflectance products that have undergone orthorectification, radiometric calibration, atmospheric correction, and cloud removal. To meet research needs, a total of 10 spectral bands were selected, namely B2 (blue), B3 (green), B4 (red), B5 (red edge 1), B6 (red edge 2), B7 (red edge 3), B8 (near infrared), B8a (narrow near infrared), B11 (shortwave infrared 1), and B12 (shortwave infrared 2). To achieve consistency in spatial resolution, all bands with an original resolution of 20 m were resampled to a spatial resolution of 10 m through nearest neighbor interpolation. Finally, a multispectral dataset of uniform scale was constructed to ensure the accuracy and reliability of subsequent analysis.

### 2.3. Cultivated Land Field Data

To fulfill the research requirements for intelligent extraction of cultivated land field, this study constructed a specialized high-resolution remote sensing image dataset. The dataset utilized sub-meter Jilin-1 satellite imagery acquired during July–August 2024 as the primary data source, supplemented by Gaofen-2 (GF2) and Gaofen-7 (GF7) satellite imagery for specific areas within Xiangfu District and Weishi County of Kaifeng City. Ground-truth labels were generated through meticulous manual visual interpretation, with precise vectorization delineation of farmland boundaries within the study area. To construct samples suitable for deep learning models, the original imagery and corresponding vector labels were uniformly processed into 512 × 512 pixel image tiles during data preprocessing. To ensure continuity of ground feature information and augment sample quantity, a 156-pixel overlap was established between adjacent tiles, yielding a total of 12,350 valid samples. Finally, the sample set was randomly partitioned into training and validation sets at a 7:3 ratio to support subsequent model parameter learning and generalize performance evaluation, respectively.

### 2.4. Sample Data

In this study, data from two scales were used to validate classification results and cropland area estimates. A total of 2252 ground sample points were collected, including wheat and garlic samples from field surveys with handheld GPS devices and samples for buildings, bare land, and water bodies from visual interpretation of high-resolution imagery on the Google Earth Engine (GEE) platform. The dataset was stratified into a training set (1576) and a validation set (676) at a 7:3 ratio using GEE’s built-in “ee.FeatureCollection.stratifiedSample” function, which maintained class proportions based on labels to address underrepresentation of minority classes (e.g., “other land” with 104 samples). This stratification was essential due to the phenotypic similarities between wheat and garlic, driven by overlapping phenological periods and similar spectral/textural signatures [3], enabling the GEE-based Random Forest model to reduce bias and improve generalization. Detailed sample distributions are provided in Table 2.

### 2.5. Feature Variables

To fully utilize multi-source remote sensing data, this study constructed a comprehensive variable set comprising 28 features across four major categories: polarization, spectral, vegetation indices, and texture (detailed in Table 3). The feature set first incorporated VV and VH polarization backscattering coefficients from Sentinel-1, which are sensitive to ground feature structure. Subsequently, multi-dimensional information from Sentinel-2 was integrated, including not only 10 original spectral bands and multiple vegetation indices designed to enhance vegetation growth characterization (such as NDVI, EVI, etc.) but, more critically, spatial texture features were introduced. The incorporation of texture features aims to effectively alleviate the prevalent “same object with different spectra, different objects with same spectra” problem in remote sensing imagery, supplementing the spatial arrangement patterns lacking in pure spectral information, reducing salt-and-pepper noise in classification results, and thereby improving model accuracy and robustness. Specifically, these texture features were extracted from the near-infrared band (B8) using the Gray-Level Co-occurrence Matrix (GLCM) method. To retain key information while avoiding data redundancy, four most representative indicators were ultimately selected: variance, entropy, energy, and homogeneity.

### 2.6. DexiNed

**Figure 3:**
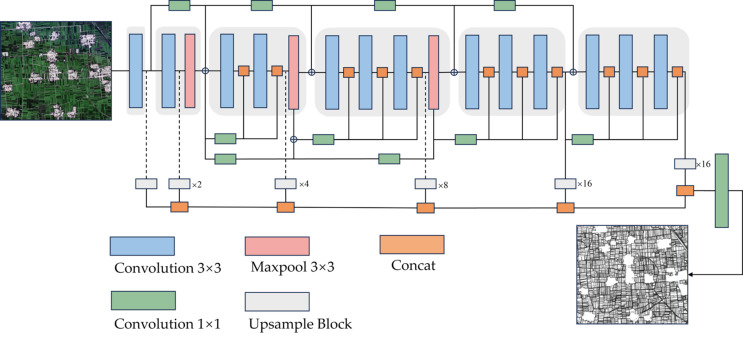
Edge Detection Network (DexiNed) Network Structure.

To achieve high-precision extraction of cultivated land field boundaries, this study employed an edge detection model based on DexiNed (Dense Extreme Inception Network for Edge Detection). As shown in Figure 3, DexiNed addresses edge detail information loss in traditional deep networks through two key innovations: Dense Blocks with skip connections that maximize information flow and alleviate gradient vanishing, and multi-scale feature fusion that extracts side outputs from different network depths, enabling simultaneous capture of both coarse contours and fine textures for comprehensive edge predictions.

Furthermore, to address the challenge of severe positive/negative sample imbalance (i.e., edge versus non-edge pixels) commonly present in edge detection tasks, this study adopted Weighted Binary Cross-Entropy (WBCE) as the loss function, formulated as follows. By introducing weight coefficients to increase the proportion of edge pixels in loss calculation, the model is guided to focus more on learning edge details during training, thereby effectively improving detection accuracy:

(1) $$L_{WBCE}=-\frac{1}{N}\sum_{i=1}^{N}\left[\omega \cdot y_{i}\log({\hat{y}}_{i})+(1-y_{i})\log(1-{\hat{y}}_{i})\right]$$

where N represents the total number of pixels; $y_{i}$ denotes the true label of the i pixel (0 or 1, indicating non-cultivated land or cultivated land); $y_{i}$ represents the predicted value of the i pixel; and ω is the weight for the positive class (cultivated land).

DexiNed model training parameters were systematically determined through empirical validation and established deep learning practices for edge detection tasks. The configuration was optimized as follows: epochs = 100, determined based on validation loss convergence patterns observed during preliminary experiments; batchSize = 8, selected to balance computational efficiency with GPU memory constraints when processing 512 × 512 input tiles; learningRate = 1 × 10^−4^, following Soria et al. [22] for edge detection applications; optimizer = Adam, chosen for its effectiveness in handling sparse gradient scenarios; and inputSize = 512 × 512, designed to provide adequate spatial context for agricultural field boundary detection.

### 2.7. Feature Optimization

This study employed the Random Forest Recursive Feature Elimination (RF-RFE) algorithm for feature optimization to select the optimal feature subset [20,23]. This method utilizes the Random Forest (RF) model as the basis for evaluating feature importance, where the assessment leverages the prediction error rate of out-of-bag (OOB) data to obtain robust measurements. Subsequently, the algorithm initiates a recursive elimination process, systematically removing one feature with the lowest current importance in each iteration. To determine the optimal feature subset size, 10-fold cross-validation was embedded in the research process, with model classification accuracy serving as the performance evaluation metric, thereby automatically identifying the feature quantity that performs best in cross-validation while ensuring that the finally selected feature combination contains no fewer than 8 features. The advantage of the RF-RFE algorithm lies in its systematic integration of Random Forest’s precise feature importance assessment, systematic screening through recursive elimination, and the robustness of cross-validation, effectively reducing data redundancy and noise while preserving the most critical information and enhancing model generalization capability.

### 2.8. Random Forest

RF is an ensemble algorithm consisting of decision trees operating in parallel, where results are determined through voting or averaging, endowing the overall model with high accuracy and generalization performance as well as good stability, making it widely applied in remote sensing image classification [16]. As a supervised learning method, RF relies on labeled training data to discriminate between land cover classes while maintaining computational efficiency and noise resistance.

In this study, the optimized feature subset was integrated with Sentinel-1/2 imagery on the Google Earth Engine platform to extract garlic cultivation areas. Hyperparameter optimization was performed using grid search on the training set to prevent overfitting and ensure robust model performance [16]. The parameter search space included numberOfTrees (50–200), variablesPerSplit (2–5), and minLeafPopulation (1–10), with Overall Accuracy as the selection criterion. The final configuration was set as numberOfTrees = 150, variablesPerSplit = 3, and minLeafPopulation = 4.

### 2.9. Evaluation Criteria

To systematically validate the accuracy of the classification results, this study constructed a comprehensive evaluation system based on a pixel-count confusion matrix. A series of standard evaluation metrics were calculated to quantitatively assess the model’s performance. The confusion matrix accurately quantifies the classification results by comparing model predictions with the ground truth data. Its structure is defined as shown in Table 4.

Based on the confusion matrix, the following evaluation metrics were calculated.

Overall Accuracy quantifies the proportion of correctly classified pixels across all categories and is calculated as

(2) $$OA=\frac{TP+TN}{TP+TN+FP+FN}$$

User’s Accuracy, also known as Precision, quantifies the reliability of prediction results and represents the proportion of pixels classified as positive that are actually positive. The formula is expressed as

(3) $$UA=\frac{TP}{TP+FP}$$

Producer’s Accuracy, also known as Recall or Sensitivity, quantifies the completeness of category identification and represents the proportion of actual positive pixels correctly identified by the model. It is expressed as

(4) $$PA=\frac{TP}{TP+FN}$$

The F1-score represents the harmonic mean of Precision (UA) and Recall (PA), comprehensively balancing commission and omission errors to provide a unified measure reflecting both aspects of model performance:

(5) $$F1\text{-}score=2\cdot \frac{UA\cdot PA}{UA+PA}$$

The Kappa coefficient provides a robust performance measure that eliminates the influence of class imbalance and chance agreements, offering a more objective assessment than simple accuracy. It is expressed as follows:

(6) $$k=\frac{p_{o}-p_{e}}{1-p_{e}}$$

where $p_{o}$ represents the observed overall accuracy, and $p_{e}$ represents the hypothetical probability of chance agreement, calculated as

(7) $$P_{e}=\frac{\left(TP+FN)(TP+FP)+(FN+TN)(TN+FP\right)}{{\left(TN+TP+FN+FP\right)}^{2}}$$

In addition to the quantitative assessment based on confusion matrices, this study incorporated field sampling in representative areas to qualitatively examine the spatial distribution consistency of classification results. This comprehensive methodology ensures both statistical rigor and practical validation of model performance.

### 2.10. Workflow of the Study

**Figure 4:**
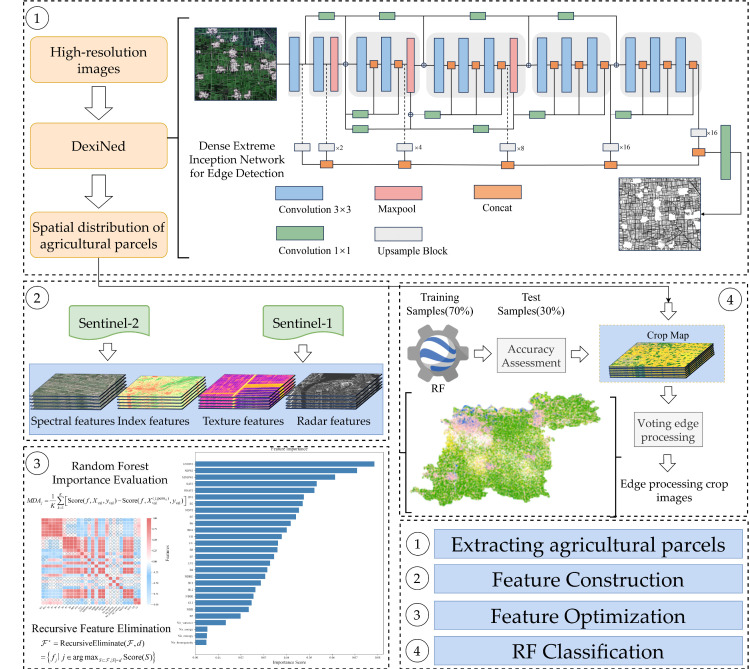
Workflow of the study.

To achieve precise crop-type identification and mapping at regional scales, the research workflow comprises four core stages: agricultural field extraction, feature construction, feature optimization, and Random Forest classification (see Figure 4 for the methodological workflow). The agricultural field extraction phase employs high-resolution imagery to train the DexiNed deep learning architecture, which performs edge detection through sequential processing of convolutional layers, max-pooling operations, and upsampling modules to generate vectorized field boundaries. In the feature construction stage, spectral features, vegetation indices, textural characteristics, and polarimetric backscatter coefficients are systematically extracted from Sentinel-2 optical and Sentinel-1 SAR data, establishing a multi-dimensional feature space. The feature optimization phase utilizes Random Forest Feature Importance analysis to rank features by significance, followed by Recursive Feature Elimination to identify optimal feature subsets that maximize classification performance. During the Random Forest classification stage, labeled samples are divided into 70% training and 30% testing sets, with the optimized features training RF classifiers to generate preliminary pixel-level crop distribution maps. These outputs are refined through spatial constraint optimization, where field vector boundaries enforce majority voting within each field unit, ultimately producing high-precision crop distribution maps for agricultural monitoring applications.

## 3. Results

### 3.1. Optimized Hyperparameter Results for Random Forest

**Figure 5:**
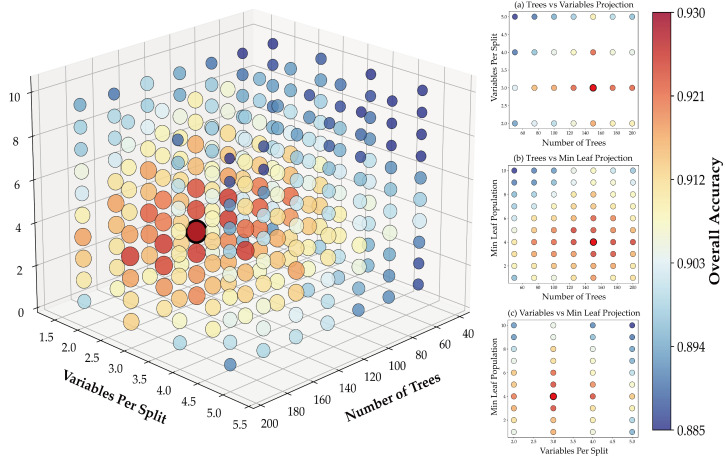
Grid Search results for Random Forest hyperparameters.

As illustrated in Figure 5, the three-dimensional parameter optimization visualization provides comprehensive insights into Random Forest model performance across diverse hyperparameter configurations for precision garlic crop identification. Systematic analysis reveals that tree quantity demonstrates a monotonic positive relationship with overall accuracy within the 50–200 range, exhibiting performance saturation beyond 150 trees consistent with diminishing marginal returns in ensemble learning theory. The variables-per-split parameter exhibits optimal performance within the 3–4 range, where configurations below this threshold induce underfitting due to insufficient feature diversity, while values exceeding this range precipitate overfitting through excessive model complexity. The minimum leaf population parameter achieves optimal bias–variance tradeoff at 3–5 nodes, with lower values introducing overfitting risk despite enhanced detail capture, and higher values potentially compromising model expressiveness. Through rigorous grid search optimization coupled with k-fold cross-validation, the optimal hyperparameter configuration was empirically determined as numberOfTrees = 150, variablesPerSplit = 3, and minLeafPopulation = 4.

### 3.2. Multi-Feature Optimization Results

**Figure 6:**
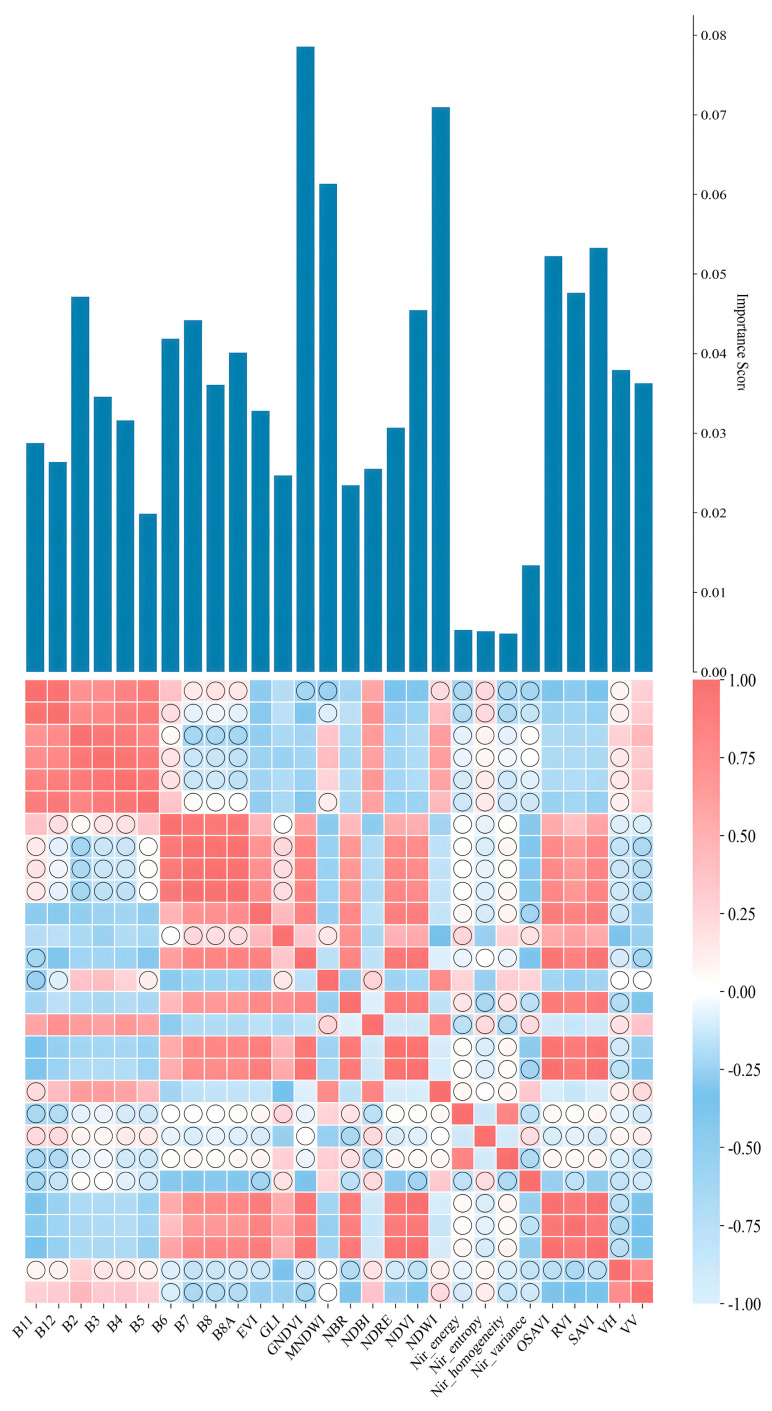
Feature importance and spatial heat map correlation analysis.

A systematic importance assessment was conducted on the 28 pre-selected multi-source remote sensing features, and Recursive Feature Elimination (RFE) was employed to optimize the feature set for constructing the most suitable feature subset for garlic identification, with the specific selection process illustrated in Figure 6. The feature importance analysis revealed significant disparities in the contributions of different feature types to the classification task. Among all candidate features, vegetation indices exhibited the highest discriminatory power, with the GNDVI feature achieving the highest importance score (0.078), closely followed by NDVI (0.072), thereby confirming the central role of vegetation indices in expressing crop spectral characteristics. Beyond vegetation indices, features including OSAVI, SAVI, and the radar polarimetric features VH and VV all attained importance scores exceeding 0.05, indicating their significant contribution to distinguishing garlic from other land cover types. In contrast, the original spectral band features (B1–B12) generally yielded lower importance scores, mostly distributed within the 0.005–0.04 range; nevertheless, certain bands (e.g., B2, B6, B7, B11) were retained in the optimal feature subset due to their unique spectral response characteristics.

Feature correlation analysis further elucidated the inherent relationships and information redundancy among multi-source features. The correlation heatmap revealed high correlations primarily concentrated in several regions: significant positive correlations were observed between spectral bands B1 and B4, manifesting as distinct red block structures in the heatmap; similarly strong positive correlations characterized bands B8A–B11. Vegetation indices NDVI, GNDVI, OSAVI, and SAVI exhibited high mutual correlation, forming a concentrated cluster of red cells. Additionally, certain vegetation indices showed moderate negative correlations with the visible bands (B2–B4), corresponding to blue regions in the heatmap. In contrast, low correlations were predominantly evident in the following aspects: most texture features (statistical measures including mean, standard deviation, and entropy derived from infrared bands) displayed weak correlations with spectral bands, with coefficients largely within the range of −0.25 to 0.25, indicated by circular markers in the heatmap; radar features VV and VH also exhibited low correlations with spectral features; furthermore, weaker correlations were observed between certain vegetation indices, such as EVI, and the near-infrared bands B8 and B8A, similarly marked by circles. These low inter-feature correlations strongly indicate substantial complementarity and independence in their information representation.

**Figure 7:**
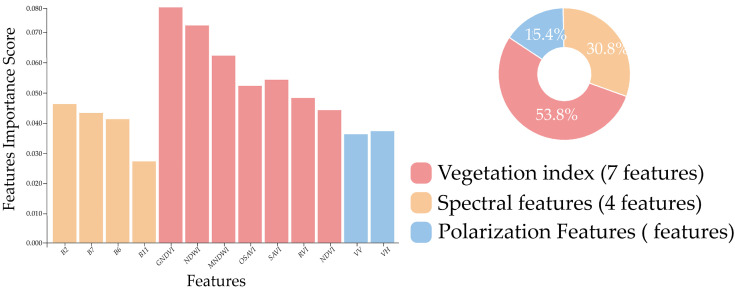
Optimal feature subset.

Based on the feature optimization results, 13 optimal features were selected from the original 28 features for subsequent classification tasks, with their importance distribution and composition illustrated in Figure 7. Among the selected features, vegetation indices predominated, accounting for 53.8% of the total importance contribution through seven features (GNDVI, NDWI, MNDWI, OSAVI, SAVI, RVI, and NDVI). The bar chart further reveals significant internal variations, with GNDVI, NDWI, and MNDVI securing the top three importance scores among all features, underscoring their critical value in the classification task. Spectral features followed, comprising four original bands (B2, B7, B6, and B11) and collectively contributing 30.8% to the total importance. Within this group, B2 (blue band) exhibited the highest individual score (0.045), significantly exceeding other spectral bands—a result closely linked to the unique spectral response characteristics of garlic leaves in the blue region. Bands B7, B6, and B11 demonstrated relatively balanced importance scores (0.038, 0.035, and 0.032, respectively), indicating the supplementary role of red-edge and shortwave infrared (SWIR) bands in characterizing garlic spectral features. Although radar polarimetric features contributed a comparatively lower share (15.4%), their unique backscatter information provided indispensable structural feature support for the classification model. The importance scores of VV and VH were 0.028 and 0.025, respectively, with minimal difference between them, suggesting similar discriminatory value for vertical and horizontal polarization information in garlic identification. Despite their lower individual rankings, the low correlation (r < 0.3) between radar features and optical features ensured their independent contribution within the multi-source data fusion framework, delivering essential complementary information for enhancing classification accuracy.

### 3.3. Random Forest Classification Results

Classification experiments were conducted before and after feature optimization to evaluate the impact of feature selection on classification performance, with comparative accuracy results presented in Table 5. Prior to feature optimization, the model achieved an Overall Accuracy (OA) of 0.91 and a Kappa coefficient of 0.8654. Following the application of the feature optimization strategy, the model’s overall classification performance demonstrated significant improvement: OA increased to 0.93 (a gain of 0.02), and the Kappa coefficient rose to 0.8857 (an increase of 0.0203). Regarding individual land cover classes, all accuracy metrics (User’s Accuracy—UA, Producer’s Accuracy—PA, F1-score) showed improvements across classes with the exception of Water, whose PA remained unchanged. For the Wheat class, UA, PA, and F1-score each increased by 0.01. For the core target class, Garlic, UA, PA, and F1-score reached 0.89, 0.90, and 0.90, respectively, representing increases of 0.01, 0.03, and 0.02, respectively, compared to the pre-optimization results. Among other classes, the Others class exhibited increases of 0.04, 0.07, and 0.06 in UA, PA, and F1-score, respectively; the Building class showed gains of 0.06, 0.01, and 0.03, respectively, while the Water class also achieved increases of 0.03 in UA and 0.02 in F1-score.

**Figure 8:**
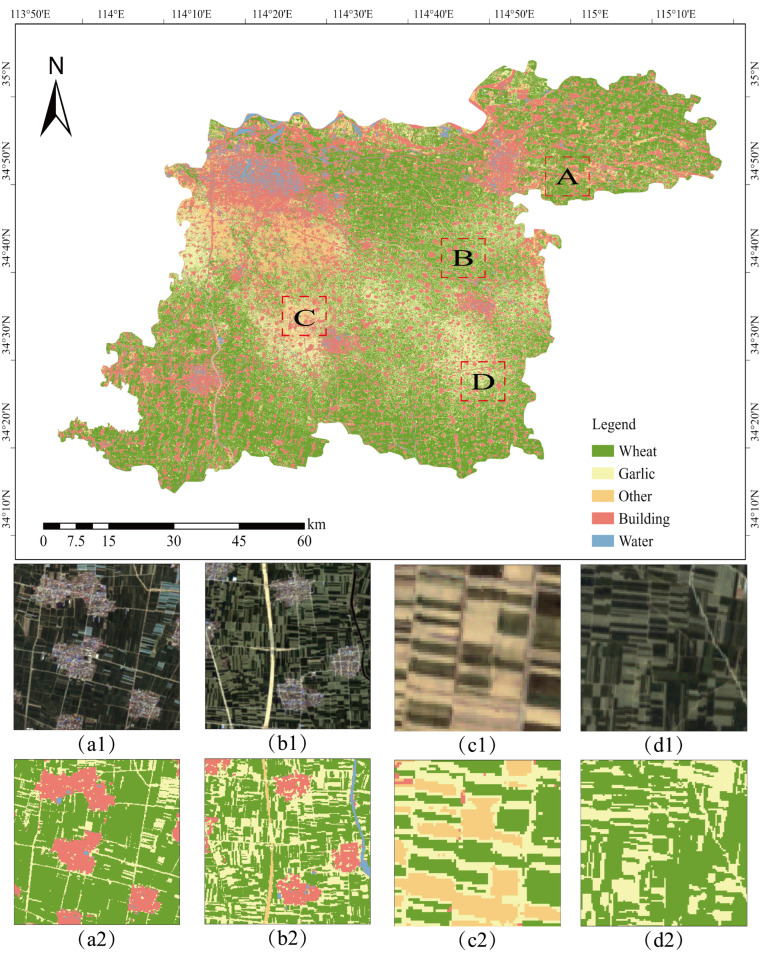
Random Forest classification results. (**a1**–**d1**) Sentinel-2 true-color composite imagery showing different agricultural landscape types for regions A–D; (**a2**–**d2**) corresponding classification results displaying wheat, garlic, buildings, and other land cover distributions.

The Random Forest classification model constructed with the optimal feature combination achieved precise identification of garlic cultivation areas within the study region, with the classification result presented in Figure 8. This map comprehensively displays the spatial distribution patterns of five major land cover types—wheat, garlic, buildings, water bodies, and other features—accurately reflecting their distribution characteristics and spatial structure at the macro scale. Overall, garlic cultivation areas were predominantly concentrated in the central and southern parts of the study area, exhibiting relatively contiguous distribution patterns that align closely with the regional agricultural structure and land use configuration. Wheat cultivation areas were intermingled spatially with garlic fields, forming a typical crop rotation pattern; while the spectral similarity between these two crops posed classification challenges, the model effectively distinguished them. Building areas, primarily located in the northern and eastern sectors, showed distinct clustered distributions consistent with actual urban and rural settlements. Water bodies, including rivers, lakes, and reservoirs, were clearly delineated with linear and areal features. Detailed analysis of representative sub-regions revealed the following: Area A accurately captured the boundary between dense building zones and farmland, clearly delineating the spatial differentiation between urban built-up areas and agricultural land; Area B demonstrated successful identification and spatial localization of multiple intermixed features (wheat, garlic, buildings, water) within a complex agricultural landscape; Areas C and D, as core cultivation zones, effectively reproduced the strip-like and block-like spatial textures of mixed wheat and garlic, validating the model’s capability to distinguish spectrally similar crops. However, the pixel-based classification approach resulted in noticeable salt-and-pepper noise in localized areas, manifested as spatial fragmentation of land field and internal class inconsistency, with scattered misclassified pixels within otherwise homogeneous areas—particularly prominent in wheat and garlic fields—which adversely affected the visual quality and practical utility of the classification results.

### 3.4. Agricultural Land Boundary Extraction Based on Deep Learning

**Figure 9:**
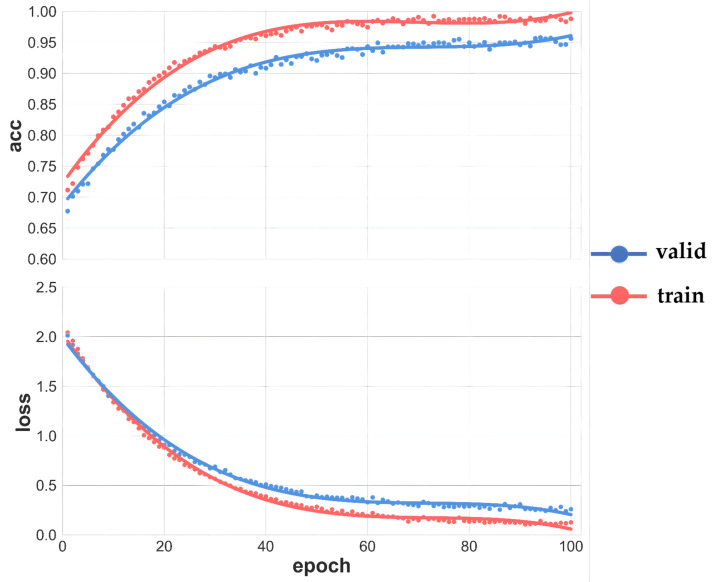
DexiNed model training accuracy.

The DexiNed model was trained using the constructed sample dataset, with its training process and classification performance illustrated in Figure 9. The training curves demonstrate favorable convergence characteristics throughout the process. In terms of accuracy, both training and validation set accuracies progressively increased from initial values of approximately 0.70, stabilizing after approximately 40 epochs; the training accuracy ultimately converged to nearly 0.99, while the validation accuracy stabilized at around 0.95, maintaining a reasonable gap that indicates the absence of significant overfitting and demonstrates robust generalization capability. Regarding the loss function, both training and validation losses decreased rapidly from initial values of approximately 2.0, exhibiting a substantial decline within the first 20 epochs before gradually stabilizing, with the training loss eventually converging to approximately 0.25 and the validation loss stabilizing at approximately 0.15.

**Figure 10:**
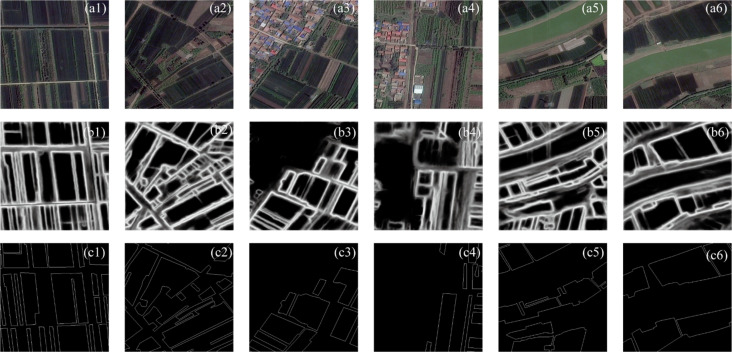
Comparison of farmland field extraction with different landform types. (**a1**–**a6**) Input satellite imagery of different landscape types; (**b1**–**b6**) DexiNed edge detection results; (**c1**–**c6**) ground truth boundary labels.

The DexiNed edge detection network demonstrated excellent performance in agricultural field boundary extraction tasks, as clearly shown in the six comparative result groups presented in Figure 10, highlighting its capability across diverse land cover types and complex scenarios. In pure cultivated areas (a1,a2), the model successfully identified precise boundaries of regular farmland field, extracting continuous and complete edge lines that accurately delineated field geometry and spatial extent. Within mixed building–cropland areas (a3,a4), it effectively distinguished boundaries between artificial structures and natural farmland; despite complex interfaces and spectral confusion, the edge detection results accurately located division lines between different land cover types. For mixed water body–cropland regions (a5,a6), the model correctly identified land–water boundaries, with detection results exhibiting high spatial consistency against ground truth field labels (b1–b6). Quantitative accuracy assessment based on 3705 independent test images (512 × 512 pixels) revealed that DexiNed achieved a User’s Accuracy of 93.22%, Producer’s Accuracy of 95.90%, and an F1-score of 94.16% (Table 6), with all comprehensive performance metrics exceeding 93%, fully validating its reliability and accuracy for agricultural field boundary extraction. The model’s output edge detection results (c1–c6) showed high spatial congruence with manually annotated field boundaries, featuring clear, continuous lines and good geometric precision that faithfully captured true field shapes. In practical extraction, the method exhibited certain commission and omission errors when processing complex agricultural field characterized by elongated shapes, narrow configurations, or ambiguous boundaries. Nevertheless, the boundaries extracted by DexiNed maintained high completeness, thereby providing precise and reliable spatial extents and boundary constraints for subsequent fine-scale agricultural applications such as field-level crop type identification and cultivation area statistics.

### 3.5. Optimization of Classification Results Based on Field Constraints

**Figure 11:**
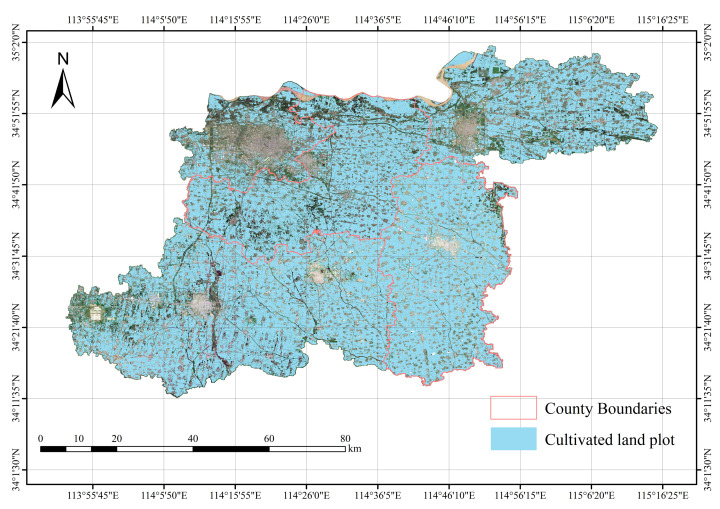
Henan cultivated land field.

To achieve crop attribute assignment at the field scale, the study first performed vectorization processing on the cultivated land field boundaries extracted by the DexiNed network, generating vector polygons covering all arable land field in Kaifeng City, Henan Province, as illustrated in Figure 11, where Xiangfu and Qi counties—highlighted in red—represent the primary garlic cultivation areas.

**Figure 12:**
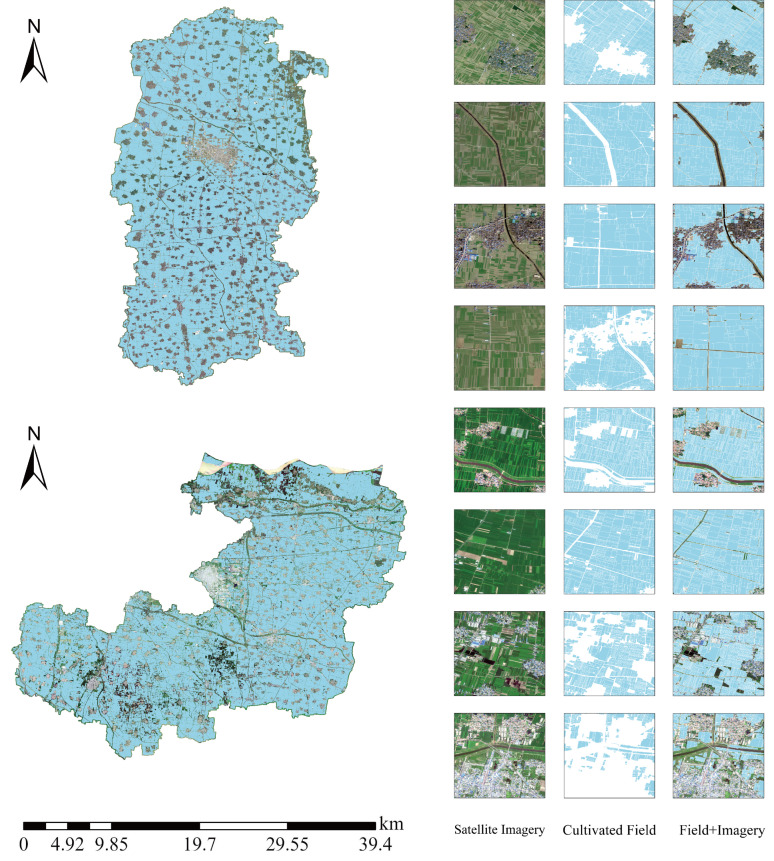
Distribution of cultivated land field in major planting areas.

As comprehensively illustrated in Figure 12, this map presents the refined cultivated land field vector polygons generated by the DexiNed network for Qi County (upper-left) and Xiangfu District (lower-left)—the primary garlic cultivation areas in Kaifeng City, Henan Province. The spatial distribution demonstrates that the method successfully achieved complete, seamless coverage of cultivated field across the study area, as shown by the light-blue polygons, which accurately exclude non-cultivated land (e.g., urban built-up areas, water bodies) and completely delineate the fundamental units of agricultural production zones. For qualitative and quantitative accuracy assessment, the right side of the figure details extraction results for multiple typical fields within key cultivation zones in a matrix format comprising three columns: original high-resolution satellite imagery, extracted cultivated field vectors, and a verification overlay (Field + Imagery). Detailed examination of these samples reveals that the extracted field boundaries not only align macroscopically with farmland regions in the imagery but also precisely capture true field geometry at the micro-scale while maintaining topological integrity among adjacent fields. The method exhibits excellent segmentation performance and boundary localization accuracy across diverse field types—including contiguous rectangular fields, fragmented irregular fields, and complex fields along roads or canals. The final overlay images clearly demonstrate high-fidelity spatial correspondence between extracted vectors and actual field edges, thereby providing a high-quality, reliable field-level geospatial vector base for subsequent precise crop attribute assignment.

**Figure 13:**
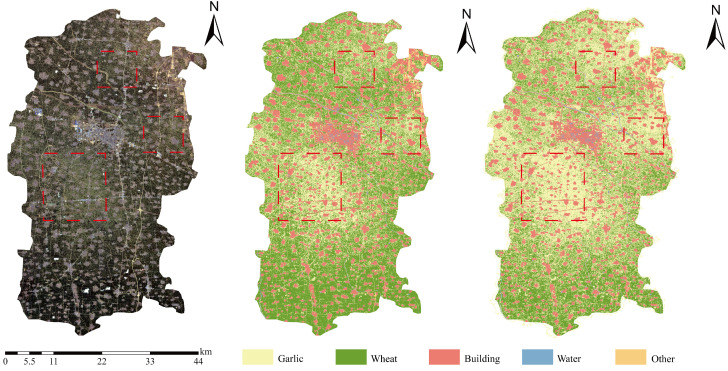
Qixian field optimization results.

To address the pervasive issue of salt-and-pepper noise in pixel-level classification, this study innovatively introduced field boundaries as spatial constraints for post-processing optimization of preliminary classification results, as illustrated in Figure 13. This strategy leverages the practical agricultural principle that a single crop type is typically cultivated within an individual field; through a majority voting mechanism that quantifies the pixel count distribution per class within each field, the dominant crop category is assigned to the entire field, enabling effective conversion from pixel-level to field-level classification. The application of field-constrained optimization yielded significant improvements: the pre-optimized Random Forest classification exhibited characteristic salt-and-pepper noise, with fragmented land cover patches, poor spatial continuity, and abundant isolated pixels and fine speckles—particularly in cropland areas—severely compromising the results’ practical utility and visual quality. Post-optimization effectively suppressed these issues, successfully aggregating fragmented pixels of identical classes into intact, homogeneous patches; intra-field class assignments became uniform, with boundaries clear and regular, rendering the spatial distribution patterns of key crops (e.g., garlic in yellow, wheat in green) more distinct and accurate. Consequently, the optimized classification better reflects actual agricultural characteristics. Validation within primary garlic cultivation areas of Qi County and Xiangfu District, via comparison of the complete processing chain (original Sentinel-2 imagery, field vectors, Random Forest classification, constrained optimization result, and final clipped product), clearly demonstrated progressive refinement: while the initial classification broadly identified land cover classes, it suffered from severe spatial noise—manifested as isolated pixels and speckles within field and blurred boundaries. In contrast, the constrained optimization substantially enhanced visual quality and spatial consistency, effectively eliminating noisy pixels, unifying intra-field class assignments, and regularizing boundaries.

**Figure 14:**
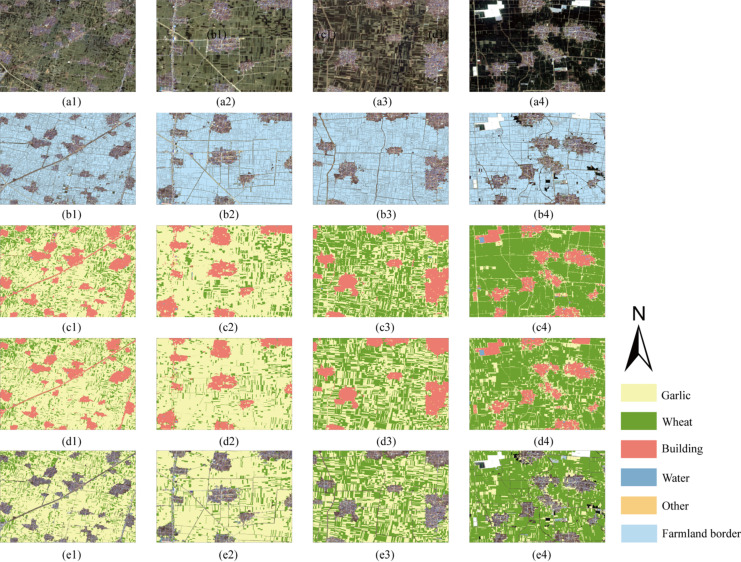
Local results of field optimization. (**a1**–**a4**) Sentinel-2 imagery; (**b1**–**b4**) vectorized field boundaries extracted by DexiNed network; (**c1**–**c4**) Random Forest pixel-level classification results; (**d1**–**d4**) field-constrained optimization; (**e1**–**e4**) final field-level crop maps.

Figure 14 visually demonstrates the effectiveness of the proposed field optimization strategy across five representative areas, systematically presenting the complete processing workflow and performance enhancement through comparison of five image types: (a) original Sentinel-2 imagery, (b) field vector data overlaid on Sentinel imagery, (c) Random Forest (RF) classification results, (d) classification maps optimized with constraint rules, and (e) final products generated by clipping optimized results using field data as vector boundaries. The original Sentinel-2 imagery column (a1–a4) clearly reveals the complex and diverse land cover conditions within the study area, including mixed distributions of dense buildings and farmland (e.g., a1), agricultural zones with regular fields interwoven with road networks (e.g., a2), and complex agricultural landscapes with spatially adjacent crop types (e.g., a3, a4). The field overlay column (b1–b4) provides a crucial spatial constraint framework for subsequent classification by precisely delineating each field’s geometric extent and boundary morphology, establishing the foundation for field-based optimization. While the RF classification results (c1–c4) broadly distinguish land cover classes, they exhibit severe salt-and-pepper effects in spatial representation: fields contain abundant isolated pixels and fine speckles, causing fragmented distributions of garlic and wheat; boundaries appear blurred and jagged, with significant class confusion at field junctions and edges. In contrast, the optimized classification maps (d1–d4) show substantial improvements in visual quality and spatial consistency: constraint rules effectively eliminate most noise, coalescing fragmented pixels of identical classes into smooth, homogeneous patches that ensure intra-field class uniformity; boundaries are reshaped into clear, regular outlines that accurately trace field contours, preserving geometric integrity for both large regular fields and irregular small fields. The final vector-clipped products (e1–e4), generated by precisely clipping optimized results using field data as spatial templates, further ensure strict adherence to actual field boundaries. This achieves perfect spatial alignment between raster classification outputs and vector field data, producing definitive garlic identification products with clear boundaries, internal homogeneity, and strong spatial integrity—thereby establishing a high-quality data foundation for subsequent agricultural applications and decision support.

## 4. Discussion

### 4.1. Methodological Innovation Through Technical Integration

Although this study employs established techniques—DexiNed for edge detection, RF-RFE for feature optimization, and Random Forest for classification—the primary innovation lies in their systematic integration for agricultural applications [24]. This integrated framework addresses limitations when methods are applied independently: DexiNed alone lacks crop-specific optimization, RF-RFE cannot address spatial fragmentation, and Random Forest suffers from salt-and-pepper noise without spatial constraints [25]. Our methodological contribution demonstrates how synergistic combination overcomes individual limitations while leveraging complementary strengths. The spatial constraint optimization using field boundaries as voting units represents a novel application, bridging pixel-level classification and agricultural production realities [26]. This integration provides a replicable framework adaptable for other crop types and geographic regions, advancing operational remote sensing for precision agriculture.

### 4.2. Integrated Technical Performance Analysis

This study implemented Random Forest Recursive Feature Elimination (RF-RFE) to select 13 optimal features from 28 candidates, significantly enhancing classification accuracy from 91% to 93%. RF-RFE’s efficacy in remote sensing crop classification is well established, with Belgiu and Drăguţ [16] confirming that Random Forest’s variable importance measures effectively identify relevant features within high-dimensional remote sensing data while reducing redundancy. Our analysis revealed vegetation indices (VIs) contributed dominantly (53.8%) to garlic identification, aligning with Yang et al.’s [5] findings on optimal feature selection. Specific VIs (GNDVI, NDVI, OSAVI) demonstrated exceptional sensitivity for discriminating garlic from co-occurring crops like winter wheat, attributable to garlic’s distinctive leaf morphology and spectral response. This observation concurs with Gao et al. [27], who identified VIs as primary contributors in GF-6-based crop classification, and Song et al. [28], who reported VIs’ superior class separability in hyperspectral feature selection. Notably, the diminished importance of raw spectral bands further substantiates VIs’ superiority. Although radar polarimetric features contributed modestly (15.4%), their low correlation with optical features (r < 0.3) ensured complementary information within the fusion framework, consistent with Trivedi et al.’s [29] findings on Sentinel-1/2 synergy. Texture features effectively mitigated spectral confusion phenomena (“same object with different spectra, different objects with same spectra”), augmenting pure spectral data with spatial arrangement patterns, corroborating Kumar and Shaikh’s [30] demonstration of texture-enhanced Random Forest performance. Comparative assessment confirms our strategy’s superiority: RF-RFE outperforms conventional feature selection methods in stability and generalization for high-dimensional multi-source data and surpasses simple feature ranking approaches [31] in handling feature interactions and nonlinear relationships.

The DexiNed network achieved superior performance in agricultural field boundary extraction, with user accuracy, producer accuracy, and F1-score of 93.22%, 95.90%, and 94.16%, respectively. This validates our hypothesis that dense skip connections and multi-scale feature fusion mechanisms effectively mitigate edge detail loss in deep networks. Compared to traditional edge detection operators (e.g., Canny, Sobel), DexiNed demonstrates enhanced adaptability to complex agricultural backgrounds. Comparative analysis confirms DexiNed substantially outperforms other deep learning edge detection methods in agricultural scenarios [21]. However, limitations manifest as occasional omissions when processing complex fields with elongated shapes or ambiguous boundaries [32]. DexiNed’s end-to-end trainability without pretrained weights provides operational advantages by reducing technical barriers to deployment [22], enabling broader regional scalability for precision agriculture.

The field-boundary-based spatial constraint optimization effectively mitigates “salt-and-pepper” noise in pixel-level classification. By enforcing single-crop dominance within individual fields, this approach transforms pixel-based results into field-level products, enhancing spatial continuity and integrity [20]. Majority voting ensures intra-field consistency and sharpens boundary delineation, yielding precise spatial distribution patterns for key crops. This optimization improves visual quality and enhances practical utility by better reflecting actual agricultural production characteristics [32]. Comparative analysis confirms optimized results exhibit superior accuracy in primary garlic cultivation zones (Qixian, Xiangfu District), providing reliable geospatial foundation for agricultural decision–support systems.

### 4.3. Methodological Limitations and Future Improvements

Notwithstanding the significant achievements of this study, several critical limitations warrant acknowledgment and future research attention. This research focuses exclusively on garlic identification in Kaifeng City’s specific geographic context, characterized by warm temperate continental monsoon climate and Yellow River alluvial plains, which raises important questions about method transferability across diverse agroecological zones. Kordi and Yousefi [33] demonstrated significant variability in crop classification across geographical regions, indicating our optimized feature set may require recalibration for different crops and environments. Future validation should systematically test the framework across multiple regions, crop types, and climatic zones. Additionally, performance evaluation with spectrally similar crops (onions, shallots, other alliums) remains essential, as spectral confusion between related species may necessitate alternative feature selection strategies.

From a computational perspective, DexiNed achieves exceptional performance but poses challenges for large-scale deployment. Training required 42 h on an NVIDIA RTX 4090 GPU, demonstrating research accessibility while indicating substantial infrastructure requirements for national applications. Processing demands scale linearly with dataset size, suggesting national implementation would require distributed computing frameworks. High-resolution imagery compounds storage demands—our dataset required 2TB storage while national applications could demand petabyte-level infrastructure. Future research should prioritize model compression, mixed-precision training, and cloud-based solutions for broader implementation.

Furthermore, while using atmospherically corrected Level-2A Sentinel data with cloud masking, residual environmental variations (seasonal illumination changes, atmospheric haze, acquisition geometry differences) may still influence spectral characteristics and feature importance rankings across temporal and geographic contexts. Enhanced robustness requires cross-seasonal validation and multi-site calibration to ensure feature stability and classification consistency for reliable operational deployment.

## 5. Conclusions

This study established an integrated technical framework for garlic identification, incorporating multi-source remote sensing data, feature optimization, deep learning-based edge detection, and spatially constrained optimization, which was successfully applied in Kaifeng City, Henan Province. The main findings and conclusions are as follows:

1. An efficient feature optimization strategy was established: Recursive Feature Elimination (RFE) selected 13 optimal features from 28 candidates, improving classification accuracy from 91% to 93%, validating the critical role of feature selection in remote sensing classification; vegetation indices contributed critically to garlic identification, with GNDVI, NDVI, and OSAVI exhibiting significantly higher importance than original spectral bands;
2. Deep learning was successfully applied for agricultural field extraction: the DexiNed edge detection network demonstrated outstanding performance in agricultural field boundary delineation, achieving User’s Accuracy, Producer’s Accuracy, and F1-score of 93.22%, 95.90%, and 94.16%, respectively, providing a reliable spatial constraint foundation for subsequent field-based crop identification;
3. An effective spatially constrained optimization strategy was proposed: the majority voting optimization method based on field boundaries successfully resolved salt-and-pepper noise in pixel-level classification, significantly enhancing the spatial continuity and integrity of classification results, rendering the final products better aligned with actual agricultural production units;
4. High-precision garlic identification was achieved: application in major garlic-producing areas of Kaifeng City demonstrated the method’s capability to accurately identify garlic cultivation zones, providing technical support for monitoring and management within the garlic industry.

Beyond these specific technical achievements, this study’s primary contribution lies not in developing new algorithms but in demonstrating the effective integration of established remote sensing and machine learning techniques to address real-world agricultural challenges. The systematic combination of deep learning edge detection, multi-source feature optimization, and spatial constraint mechanisms provides a robust, scalable framework for precision crop identification that advances beyond the limitations of individual techniques applied in isolation.

## Figures and Tables

**Table 1:** Sentinel-1/2 image data parameters.

		Center Wavelength (nm)		
Transducer	Band Name	S2A	S2B	Resolution (m)
Sentinel-1 SAR	VV	5.405 GHz 5.405 GHz		10
	VH			10
	B1	443.9	442.3	60
Sentinel-2 MSI	B2	496.6	492.1	10
	B3	560.0	559.0	10
	B4	664.5	665.0	10
	B5	703.9	703.8	20
	B6	740.2	739.1	20
	B7	782.5	779.7	20
	B8	835.1	833.0	10
	B8A	864.8	864.0	20
	B9	945.0	943.2	60
	B10	1373.5	1376.9	60
	B11	1613.7	1610.4	20
	B12	2202.4	2185.7	20

**Table 2:** Sample datasets.

Data Category	Winter Wheat	Garlic	Other Land	Building	Water	Total
Training datasets	747	417	74	245	93	1576
Verification datasets	321	180	30	106	39	676
Total	1068	597	104	351	132	2252

**Table 3:** Feature variables.

Sensor	Feature Type	Feature Variables
Sentinel-1	Polarization Features	VV
		VH
Sentinel-2	Spectral Features	B2
		B3
		B4
		B5
		B6
		B8
		B8A
		B11
		B12
	Vegetation index Features	Normalized Difference Vegetation Index (NDVI)
		Enhanced Vegetation Index (EVI)
		Soil-Adjusted Vegetation Index (SAVI)
		Green Normalized Difference Vegetation Index (GNDVI)
		Green Leaf Index (GLI)
		Optimized Soil-Corrected Vegetation Index (OSAVI)
		Ratio Vegetation Index (RVI)
		Normalized Difference Red Edge (NDRE)
		Normalized Difference Built-up Index (NDBI)
		Normalized Difference Water Index (NDWI)
		Normalized Burn Ratio (NBR)
		Modified Normalized Difference Water Index (MNDWI)
	Texture features	NIR Variance Texture Feature (Nir_variance)
		NIR Energy Texture Feature (Nir_energy)
		NIR Entropy Texture Feature (Nir_entropy) NIR Homogeneity Texture Feature (Nir_homogeneity)

**Table 4:** Confusion matrix.

		Ground Truth	
		Positive	Negative
Prediction	Positive	TP	FP
	Negative	FN	TN

Where True Positive (TP) denotes the number of pixels that actually belong to the target category and are correctly classified as such; False Positive (FP) denotes the number of pixels that do not actually belong to the target category but are erroneously classified into this category (commission error); False Negative (FN) denotes the number of pixels that actually belong to the target category but are incorrectly excluded or misclassified into another category (omission error); and True Negative (TN) denotes the number of pixels that do not belong to the target category and are correctly classified as not belonging to it.

**Table 5:** Comparison of classification performance before and after feature selection.

Classification Category	Before Feature Selection			After Feature Selection		
	UA	PA	F1-Score	UA	PA	F1-Score
Wheat	0.93	0.93	0.93	0.94 ↑0.01	0.94 ↑0.01	0.94 ↑0.01
Garlic	0.88	0.87	0.88	0.89 ↑0.01	0.90 ↑0.03	0.90 ↑0.02
Others	0.92	0.79	0.85	0.96 ↑0.04	0.86 ↑0.07	0.91 ↑0.06
Building	0.89	0.93	0.91	0.95 ↑0.06	0.94 ↑0.01	0.94 ↑0.03
Water	0.91	0.91	0.91	0.94 ↑0.03	0.91	0.93 ↑0.02
	OA = 0.91 Kappa = 0.8654			OA = 0.93 ↑0.02 Kappa = 0.8857 ↑0.0203		

↑ indicates increase after feature selection.

**Table 6:** DexiNed model classification accuracy evaluation.

Network Model	Evaluation Indicators		
	UA	PA	F1-Score
DexiNed	93.22%	95.90%	94.16%

## Data Availability

The data that support the findings of this study are available from the corresponding author upon reasonable request.
